# Advancing Renewable Fuel Production from the Macroalga *Ulva lactuca* through Integrated Optimization of Biodiesel Yield and Physicochemical Properties

**DOI:** 10.1021/acsomega.6c07288

**Published:** 2026-09-19

**Authors:** Mohammad Aliff Shakir, Muna Abdul Azeez, Xiaofeng Zheng, Mardiana Idayu Ahmad

**Affiliations:** † School of Industrial Technology, 26689 Universiti Sains Malaysia, George Town, Penang 11800, Malaysia; ‡ Renewable Biomass Transformation Cluster, School of Industrial Technology, Universiti Sains Malaysia, George Town, Penang 11800, Malaysia; § Buildings, Energy and Environment Research Group, Faculty of Engineering, 6123 University of Nottingham, Nottingham NG7 2RD, U.K.

## Abstract

Algae-derived lipids are promising renewable feedstocks for biodiesel production. However, efficient conversion requires optimization of transesterification conditions to maximize biodiesel yield. This study aimed to optimize biodiesel production from *Ulva lactuca* lipids using potassium hydroxide-catalyzed transesterification. Lipids were extracted from dried biomass using Soxhlet extraction, and Response Surface Methodology (RSM) was applied to evaluate the effects of catalyst loading, reaction temperature, and reaction time through 14 experimental runs. The developed model identified optimum conditions of 0.1 wt % catalyst loading, 75 °C, and 75 min, achieving a maximum experimental biodiesel yield of 89%. The produced biodiesel exhibited a density of 862 kg/m^3^, kinematic viscosity of 5.50 mm^2^/s, flash point of 179 °C, and acid value of 1.68 mg KOH/g. While several properties met the applicable ASTM D6751 and/or EN 14214 specifications, the acid value exceeded the specified limit, indicating the need for further improvement in fuel quality. The RSM effectively identified suitable transesterification conditions for maximizing biodiesel yield from *Ulva lactuca* lipids, supporting its potential as a nonfood feedstock for renewable fuel production. These findings demonstrate the value of process optimization in advancing *Ulva lactuca* as an alternative nonfood lipid resource for sustainable biodiesel development.

## Graphical abstract

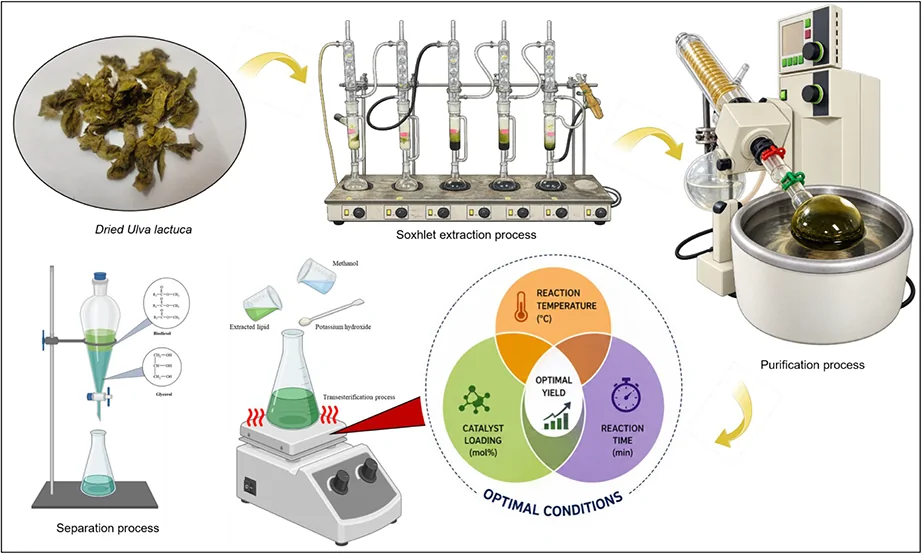

## 1 Introduction

Increasing global energy demand, fossil fuel depletion, and concerns over environmental pollution and greenhouse gas emissions have accelerated the transition toward renewable and sustainable energy systems.^1,2^ Governments and international organizations have consequently intensified efforts to promote cleaner energy technologies and reduce dependence on fossil-derived fuels.^3^ Among renewable fuel alternatives, biodiesel has emerged as a promising substitute for conventional diesel due to its renewable nature, biodegradability, and compatibility with existing diesel engines.^4^ Biodiesel may also contribute to reducing net carbon emissions and diversifying the global energy portfolio.

Conventional biodiesel production primarily relies on edible vegetable oils and animal fats, raising concerns regarding food security, land-use competition, and production costs.^5^ These limitations have encouraged the exploration of nonfood feedstocks such as algae, which offer rapid growth, high biomass productivity, and cultivation without requiring fertile agricultural land.^6^ Among macroalgal species, *Ulva lactuca* is a promising biodiesel feedstock due to its widespread availability, rapid growth, environmental adaptability, and ability to utilize nutrients from aquatic systems.^7^ Yameen et al. demonstrated the feasibility of producing biodiesel from *Ulva lactuca* lipids using a Cu-BTC@AC catalyst synthesized from residual algal biomass, providing an innovative approach for sustainable biodiesel production.^8^

Biodiesel is commonly produced through transesterification, in which lipids react with alcohol in the presence of a catalyst to produce fatty acid methyl esters (FAMEs) and glycerol as a byproduct. Due to its simplicity, economic feasibility, and high conversion efficiency, transesterification remains the most widely adopted method for biodiesel synthesis.^9^ The process can be carried out using homogeneous or heterogeneous catalysts, depending on the desired reaction conditions and product quality.^10^ Among homogeneous alkaline catalysts, potassium hydroxide (KOH) is commonly used in biodiesel production because it promotes the transesterification of triglycerides with alcohol, facilitating the formation of FAMEs. KOH provides high catalytic activity under relatively mild reaction conditions, making it suitable for efficient biodiesel production. Nevertheless, the effectiveness of the process is strongly influenced by operating parameters such as catalyst loading, reaction temperature, and reaction time. Bajwa et al. investigated the optimization of biodiesel production from a waste cooking oil-sesame seed oil blend using Response Surface Methodology (RSM) and reported that the interaction among catalyst concentration, methanol-to-oil ratio, reaction time, and stirring speed played a crucial role in maximizing biodiesel yield.^11^ Previous studies have reported that excessive catalyst loading may promote side reactions and reduce biodiesel yield, while nonoptimal reaction temperatures can adversely affect the transesterification process and limit ester formation.^12^ Therefore, optimizing the transesterification conditions is crucial for enhancing conversion efficiency and improving overall process performance.

Response Surface Methodology (RSM) has been widely applied as an effective statistical tool for optimizing biodiesel production processes. By evaluating the interactions among multiple operating variables simultaneously, RSM enables the identification of optimal process conditions while reducing the number of experimental trials required.^13^ Compared with conventional one-factor-at-a-time approaches, RSM offers a more efficient and systematic framework for process optimization. Novita et al. employed RSM to optimize biodiesel production from used palm cooking oil and reported a maximum biodiesel yield of 99.01%, highlighting the capability of RSM to identify optimal transesterification conditions.^14^ However, to the best of our knowledge, the combined application of *Ulva lactuca* lipids, KOH-catalyzed transesterification, and RSM-based optimization for biodiesel production has not been previously reported. Consequently, the interactive effects of key operating parameters such as catalyst loading, reaction temperature, and reaction time on biodiesel yield from this specific system remain insufficiently understood. This knowledge gap highlights the need for systematic optimization to establish suitable operating conditions for efficient biodiesel production from *Ulva lactuca* lipids.

Therefore, this study aimed to optimize biodiesel production from *Ulva lactuca* lipids through potassium hydroxide-catalyzed transesterification. Response Surface Methodology based on a Box-Behnken Design was employed to evaluate the effects of catalyst loading, reaction temperature, and reaction time on biodiesel yield and to determine the optimum operating conditions. Accordingly, the novelty of this study lies in the systematic optimization of KOH-catalyzed transesterification of *Ulva lactuca* lipids using RSM with emphasis on the individual and interactive effects of catalyst loading, reaction temperature, and reaction time. Furthermore, the synthesized biodiesel was characterized to assess its fuel properties in comparison with international biodiesel standards. The integration of process optimization and fuel characterization provides a comprehensive evaluation of the potential of *Ulva lactuca* as a nonfood feedstock for biodiesel production. The findings provide valuable insights into the utilization of marine macroalgae for sustainable biodiesel development and contribute to ongoing efforts toward renewable and low-carbon energy systems.

## 2 Materials and Methods

### 2.1 Sample Preparation

Fresh *Ulva lactuca* biomass was collected from coastal areas of Sabah, Malaysia. The samples were thoroughly washed with clean water to remove residual salts, sand particles, and surface contaminants. The biomass was subsequently dried in a drying oven (Memmert UF110, Germany) at 70 °C for 48 h. The dried biomass was then ground using a Bran Salzburger Grain Mill to produce a fine powder with an approximate particle size of 1 mm. The resulting powder was subsequently used for lipid extraction.

### 2.2 Lipid Extraction

Lipid extraction from dried *Ulva lactuca* biomass was performed using a Soxhlet apparatus as shown in Figure 1. A binary solvent system consisting of methanol and hexane (2:1, v/v) was prepared with a total volume of 450 mL. Subsequently, 15 g of powdered biomass was placed in a cellulose thimble and extracted at 70 °C for 7 h. Upon completion of the extraction, the solvent was removed using a rotary evaporator to recover the crude lipid fraction. The extraction process resulted in a crude lipid yield of 4.06% based on the initial dry biomass. The extracted oil was then filtered to remove residual impurities prior to transesterification.

**Figure 1:**
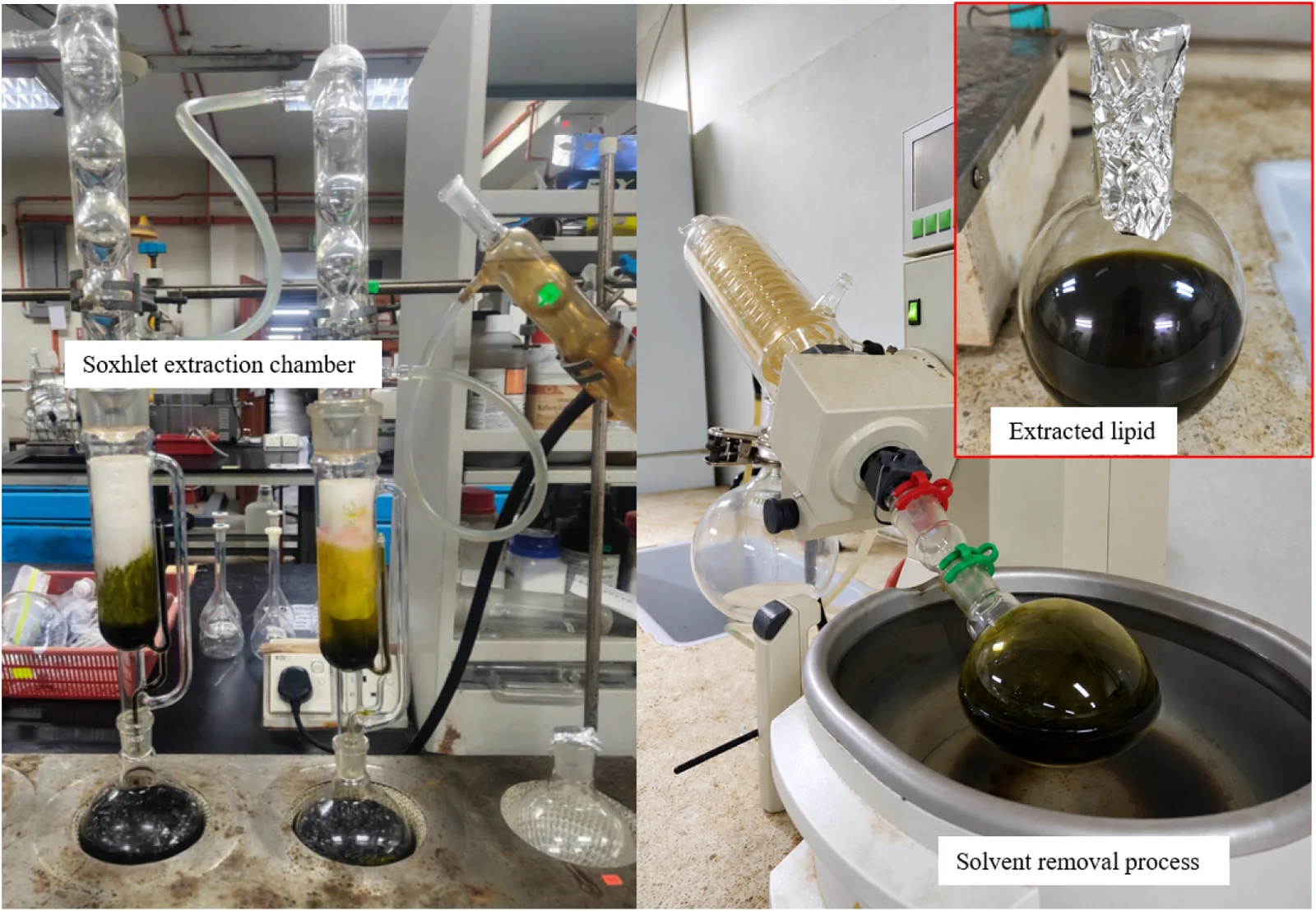
Soxhlet extraction setup used for lipid extraction from *Ulva lactuca* biomass.

### 2.3 Biodiesel Synthesis

The extracted lipid was converted into biodiesel through an alkali-catalyzed transesterification process using potassium hydroxide (KOH) as the catalyst. Preliminary experiments indicated that the extracted *Ulva lactuca* lipid exhibited relatively high viscosity, which limited effective mixing between the lipid and methanol when lower amounts of methanol were used. Therefore, 27 g of methanol was used for 2.5 g of lipid, corresponding to a methanol-to-lipid mass ratio of 10.8:1, to improve contact between the reactants during transesterification. Based on preliminary experiments, this methanol-to-lipid ratio was fixed throughout the study to minimize variation associated with alcohol availability and to allow the RSM analysis to focus on the effects of catalyst loading, reaction time, and reaction temperature. KOH was first dissolved in methanol to form potassium methoxide, which was subsequently added to the preheated lipid. The catalyst loading (0.1–1.0 wt %) was calculated based on the mass of lipid used for transesterification. The reaction was conducted in a 250 mL closed reaction vessel under continuous stirring at 300 rpm. The transesterification was conducted in a closed reaction system to minimize methanol evaporation and loss at temperatures above its normal atmospheric boiling point. Elevated temperatures were investigated based on preliminary experiments, as they improved the fluidity of the relatively viscous *Ulva lactuca* lipid and enhanced contact between the lipid and methanol. A schematic representation of the transesterification process and subsequent phase separation is shown in Figure 2. Upon completion of the reaction, the mixture was transferred into a separating funnel and allowed to stand for 5 h to facilitate phase separation. The upper biodiesel layer was separated from the lower glycerol layer and washed three times with approximately 200 mL of warm distilled water until the wash water became clear. After each washing cycle, the aqueous phase was separated from the biodiesel using a separating funnel. The washed biodiesel was subsequently dried in an oven at 100 °C until a constant weight was achieved to ensure the removal of residual moisture. The biodiesel yield was determined gravimetrically based on the mass of purified biodiesel recovered after drying relative to the initial mass of lipid used for transesterification as expressed in eq 1.

1 $$\mathrm{Biodiesel yield}\;(\%)=\frac{\mathrm{Mass of recovered biodiesel}\;(g)}{\mathrm{Initial mass of lipid}\;(g)}\times 100$$

**Figure 2:**
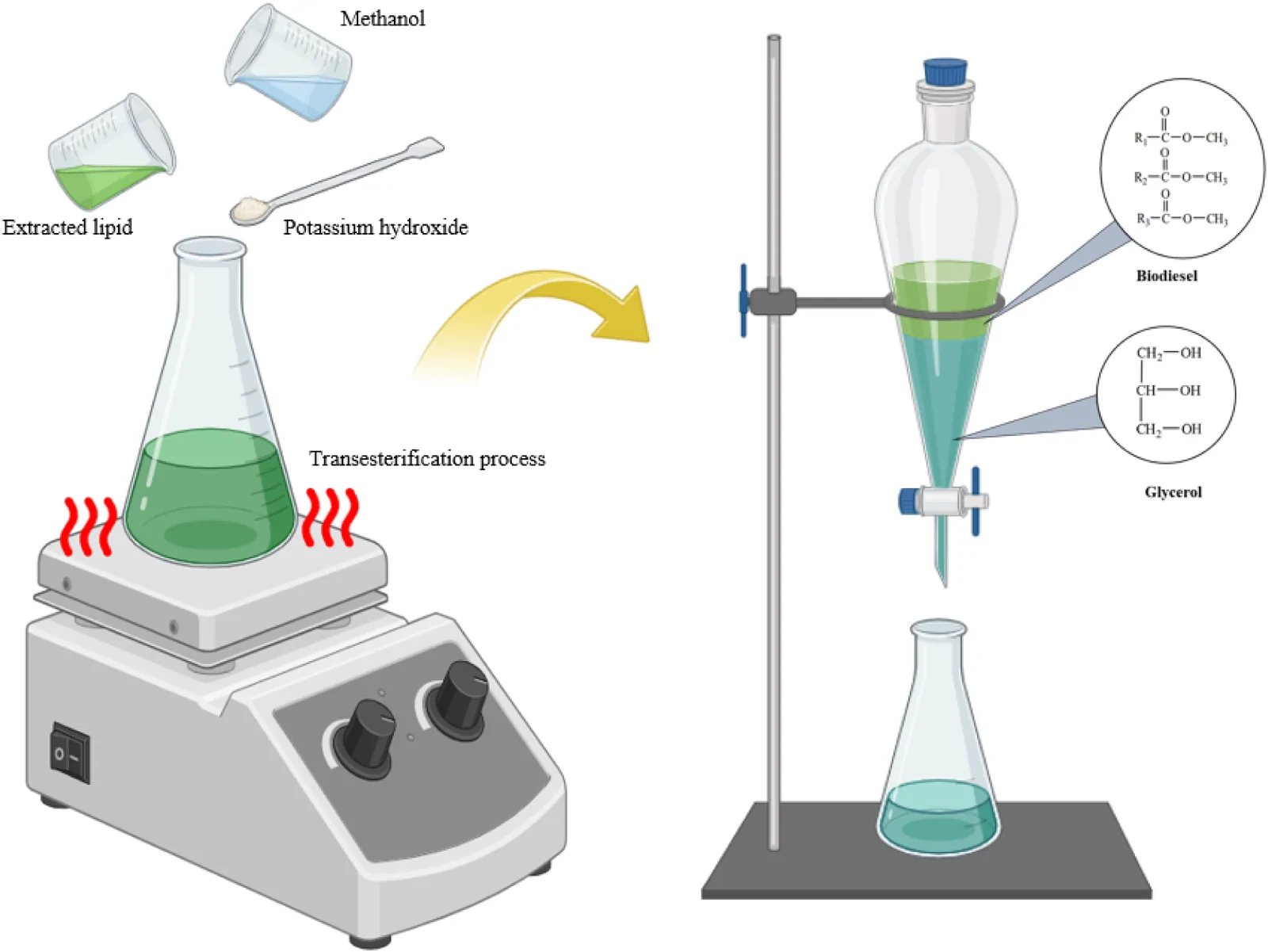
Alkali-catalyzed transesterification process for biodiesel production from *Ulva lactuca* lipid.

Accordingly, the yield reported in this study represents the recovered biodiesel product yield and should be distinguished from lipid conversion and actual FAME yield.

### 2.4 Design of Experiment

Response Surface Methodology (RSM), specifically the Box-Behnken Design (BBD), was employed to optimize the biodiesel production process. The three-factor, three-level Box-Behnken Design consisted of 14 experimental runs, comprising 12 design-point runs and two center-point runs. The objective was to determine the optimal conditions of catalyst loading (wt %), reaction time (min), and reaction temperature (°C) for maximizing biodiesel yield. These independent variables were denoted as A, B, and C, respectively. The investigated ranges of catalyst loading (0.1–1.0 wt %), reaction time (45–75 min), and reaction temperature (65–85 °C) were selected based on preliminary experiments. These preliminary trials were conducted to identify practical operating ranges that produced measurable variations in biodiesel yield and were suitable for subsequent RSM optimization. The selected ranges were subsequently assigned as the low and high levels in the Box-Behnken Design. The selected factors and their corresponding levels were summarized in Table 1. The efficiency of the transesterification process was evaluated based on biodiesel yield (*Y*_1_). The experimental design, including actual and coded values (−1, 0, and +1 representing low, medium, and high levels, respectively), together with the predicted responses, was tabulated in Table 2.

**Table 1:** Levels of the Independent Variables Range for the Transesterification Model

				Coded levels		
Factors	Factors	Symbol	Unit	Low (−1)	Medium (0)	High (+1)
A	Catalyst loading	*X*_1_	wt %	0.1	0.55	1
B	Reaction time	*X*_2_	min	45	60	75
C	Reaction temperature	*X*_3_	°C	65	75	85

**Table 2:** RSM Design for the Input Variables and Responses of Actual and Predicted Values

	Input variables			Responses yield (*Y*_1_) (%)	
Run	Catalyst (wt %) (*X*_1_)	Time (min) (*X*_2_)	Temperature (°C) (*X*_3_)	Actual value	Predicted value
1	0.55	45	85	53.8	53.75
2	0.1	75	75	89.0	87.35
3	0.1	60	65	53.4	55.00
4	0.55	45	65	43.8	42.05
5	1	45	75	63.4	65.05
6	1	60	65	66.6	66.70
7	0.55	75	65	64.2	64.25
8	1	75	75	54.6	54.45
9	0.55	75	85	43.4	45.15
10	0.55	60	75	41.8	41.80
11	0.1	45	75	63.0	63.15
12	0.55	60	75	41.8	41.80
13	1	60	85	37.4	35.80
14	0.1	60	85	78.6	78.50

### 2.5 Characterization of Biodiesel

Preliminary transesterification experiments were conducted prior to RSM optimization, and FTIR and GC-FID analyses were used at this stage to confirm the formation of fatty acid methyl esters (FAMEs) and establish the suitability of the transesterification procedure. The preliminary experiments were conducted within the same ranges of catalyst loading, reaction time, and reaction temperature subsequently investigated using RSM. Following optimization, the physicochemical properties of the recovered biodiesel product, including density, kinematic viscosity, cloud point, pour point, flash point, acid value, calorific value, and iodine number, were determined and compared with relevant biodiesel standards.

#### 2.5.1 Density

The density of the synthesized biodiesel was determined at 15 °C in accordance with ASTM D1298.^15^ Biodiesel density was calculated from the ratio of sample mass to volume using eq 2. All measurements were performed to evaluate fuel quality and compliance with biodiesel standards.

2 $$\mathrm{Density}=\frac{\mathrm{Mass}\;(m)}{\mathrm{Volume}\;(V)}$$

#### 2.5.2 Viscosity

The kinematic viscosity of the synthesized biodiesel was measured at 40 °C using a CANNON viscometer in accordance with ASTM D445.^9^ The viscosity values obtained were used to evaluate the flow characteristics of the biodiesel and its compliance with established fuel quality standards.

#### 2.5.3 Pour Point

The pour point of the synthesized biodiesel was determined in accordance with ASTM D97.^16^ Biodiesel samples were transferred into suitable test containers and cooled under controlled conditions. During the cooling process, the samples were periodically inspected to assess their flow characteristics. The temperature at which the biodiesel ceased to flow upon tilting the container was recorded as the pour point. This analysis was conducted to evaluate the low-temperature flow behavior of the biodiesel.

#### 2.5.4 Flash Point

The flash point of the synthesized biodiesel was determined according to ASTM D93.^17^ Biodiesel samples were placed in a test chamber and heated at a controlled rate. An ignition source was periodically introduced above the sample surface during heating. The lowest temperature at which the biodiesel vapors ignited momentarily was recorded as the flash point. The measurement was performed to assess the ignition characteristics and handling safety of the biodiesel fuel.

#### 2.5.5 Acid Value

The acid value of the synthesized biodiesel was determined according to ASTM D664 using a standard titration method.^18^ Biodiesel samples were titrated with 0.01 N potassium hydroxide (KOH), and the acid value was calculated using eq 3. The analysis was conducted to quantify the free fatty acid content present in the biodiesel.

3 $$\mathrm{Acid value}\;(\mathrm{mg KOH}g^{-1})=\frac{\left(V_{s}-V_{b}\right)\times N\times MW}{W}$$

where *V*_s_ is the volume of KOH used for sample titration (mL), *V*_b_ is the volume of KOH used for the blank (mL), *N* is the normality of KOH, *MW* is the molecular weight of KOH, and *W* is the sample weight (g).

#### 2.5.6 Calorific Value

The calorific value of the synthesized biodiesel was determined using a calibrated Parr 6200 Isoperibol oxygen bomb calorimeter (Parr Instrument).^19^ Prior to analysis, an appropriate amount of biodiesel sample was placed in the combustion chamber of the instrument and subjected to complete combustion under a pressurized oxygen atmosphere. The measurement was performed in triplicate, and the mean calorific value was reported in MJ/kg. The analysis was performed to evaluate the energy content of the fuel and its potential suitability as an alternative energy source.

#### 2.5.7 Iodine Value

The iodine value of the synthesized biodiesel was determined according to EN 14111 using the Wijs method.^20^ The analysis was conducted to evaluate the degree of unsaturation of the fatty acid methyl esters (FAMEs) present in the biodiesel. The iodine value was expressed as grams of iodine absorbed per 100 g of biodiesel (g I_2_/100 g).

#### 2.5.8 Cloud Point

The cloud point of the synthesized biodiesel was determined in accordance with ASTM D2500.^17^ Prior to analysis, the biodiesel sample was placed in a transparent container and cooled under controlled conditions. During cooling, the sample was periodically examined for the appearance of wax crystals. The temperature at which the first visible cloudiness appeared due to crystal formation was recorded as the cloud point. All measurements were conducted to evaluate the low-temperature flow characteristics of the biodiesel and its suitability for fuel applications.

## 3 Results and Discussion

### 3.1 Development and Analysis of Regression Model

The experimental design matrix generated by Response Surface Methodology (RSM) together with the actual and predicted biodiesel yields is presented in Table 2. A total of 14 experimental runs were performed to evaluate the effects of catalyst loading (*X*_1_), reaction time (*X*_2_), and reaction temperature (*X*_3_) on biodiesel production from *Ulva lactuca* lipids. The biodiesel yield obtained from the experiments ranged from 37.4% to 89.0%, demonstrating a substantial influence of the selected transesterification parameters on the conversion efficiency. The highest biodiesel yield of 89.0% was achieved at a catalyst loading of 0.1 wt %, reaction time of 75 min, and reaction temperature of 75 °C.

Based on the experimental data, RSM suggested a quadratic model as the most suitable model for describing the relationship between biodiesel yield and the investigated process variables. The developed coded regression equation is expressed by eq 4.

4 $$Y_{1}=41.80-7.75X_{1}+3.40X_{2}-1.85X_{3}-8.70X_{1}X_{2}-13.60X_{1}X_{3}-7.70X_{2}X_{3}+16.70X_{1}X_{1}+9.00X_{2}X_{2}+0.50X_{3}X_{3}$$

Referring to eq 4, positive coefficients indicate synergistic effects on biodiesel yield, whereas negative coefficients represent antagonistic effects. The linear coefficient of catalyst loading (*X*_1_) exhibited a negative effect on biodiesel production, suggesting that increasing catalyst concentration beyond the optimum level reduced the conversion efficiency. In contrast, reaction time (*X*_2_) showed a positive influence on biodiesel yield, indicating that longer reaction durations promoted the transesterification process within the investigated range. The interaction terms *X*_1_ *X*_2_, *X*_1_ *X*_3_, and *X*_2_ *X*_3_ all displayed negative coefficients, demonstrating that simultaneous increases in these variables may adversely affect biodiesel production.

The adequacy and significance of the developed model were further evaluated using analysis of variance (ANOVA) as presented in Table 3. The quadratic model was highly significant with an *F*-value of 77.66 and a *p*-value of 0.0004. Generally, model terms with *p*-values lower than 0.05 are considered statistically significant. Among the linear factors, catalyst loading (*X*_1_) exhibited the highest significance with a *p*-value of 0.0004 and an *F*-value of 114.68, indicating that it was the most influential parameter affecting biodiesel yield. Reaction time (*X*_2_) was also significant, with a *p*-value of 0.0093 and an *F*-value of 22.07. In contrast, reaction temperature (*X*_3_) showed a *p*-value of 0.0629, indicating that its individual effect was not statistically significant within the investigated experimental range. Nevertheless, reaction temperature was retained in the model because its interactions with catalyst loading (*X*_1_ *X*_3_, *p* = 0.0002) and reaction time (*X*_2_ *X*_3_, *p* = 0.0017) were statistically significant, indicating that its influence on biodiesel yield depended on interactions with the other process variables.

**Table 3:** Analysis of Variance (ANOVA) for Fitted Variables within the Designed Model

Source	Sum of square	Degree of freedom	Mean square	*F*-value	*p*-value
**Model**	2928.43	9	325.38	77.66	0.0004
***X***_**1**_ **-Catalyst loading**	480.50	1	480.50	114.68	0.0004
***X***_**2**_ **-Time**	92.48	1	92.48	22.07	0.0093
***X***_**3**_ **-Temperature**	27.38	1	27.38	6.53	0.0629
***X***_**1**_ ***X***_**2**_	302.76	1	302.76	72.26	0.0011
***X***_**1**_ ***X***_**3**_	739.84	1	739.84	176.57	0.0002
***X***_**2**_ ***X***_**3**_	237.16	1	237.16	56.60	0.0017
***X***_**1**_ ***X***_**1**_	892.45	1	892.45	212.99	0.0001
***X***_**2**_ ***X***_**2**_	259.20	1	259.20	61.86	0.0014
***X***_**3**_ ***X***_**3**_	0.8000	1	0.8000	0.1909	0.6847
**Residual**	16.76	4	4.19		
**Lack of fit**	16.76	3	5.59		
**Pure error**	0.0000	1	0.0000		
**Cor total**	2945.19	13			

The interaction effects between variables were also found to be significant. Among the interaction terms, *X*_1_ *X*_3_ exhibited the highest *F*-value of 176.57 and a *p*-value of 0.0002, demonstrating a strong interaction between catalyst loading and reaction temperature on biodiesel yield. Likewise, *X*_1_ *X*_2_ and *X*_2_ *X*_3_ significantly influenced the response, with *p*-values below 0.05. The quadratic terms *X*_1_ *X*_1_ and *X*_2_ *X*_2_ were also highly significant, indicating the presence of curvature effects in the response surface, whereas *X*_3_ *X*_3_ showed no significant contribution to the model. The model had four residual degrees of freedom. In addition, the two center-point runs produced identical responses, resulting in zero pure error. Consequently, a meaningful lack-of-fit assessment could not be performed for the present model. This limitation should be considered when interpreting the statistical adequacy and predictive performance of the model.

The reliability of the developed model was further evaluated using the statistical parameters summarized in Table 4. The coefficient of determination (*R* ^2^) was 0.9943, indicating that 99.43% of the variation in biodiesel yield could be explained by the developed quadratic model. The adjusted *R* ^2^ (0.9815) and predicted *R* ^2^ (0.9089) were in reasonable agreement, confirming the strong predictive capability of the model. Furthermore, the low coefficient of variation (CV) of 3.61% indicates good experimental precision and reproducibility. The adequate precision value of 29.7979 was substantially higher than the minimum recommended value of 4, demonstrating an adequate signal-to-noise ratio and confirming that the model can be effectively used to navigate the design space. Collectively, these statistical indicators suggest good model performance for predicting biodiesel yield within the investigated experimental range. However, the model should be interpreted with consideration of the limited residual degrees of freedom and the inability to meaningfully assess lack of fit.

**Table 4:** Model Fit Summaries

Summary of fit	Response *Y*_1_
**Standard deviation (SD)**	2.05
**Mean**	56.77
**Coefficient of variation (CV)**	3.61
***R*** ^**2**^	0.9943
**Adjusted-*R*** ^**2**^	0.9815
**Predicted-*R*** ^**2**^	0.9089
**Adequate precision (AP)**	29.7979

### 3.2 Response Surface Analysis of Transesterification Parameters on Biodiesel Yield

The effects of catalyst loading, reaction time, and reaction temperature on biodiesel yield were evaluated using three-dimensional response surface and contour plots generated by Response Surface Methodology (RSM) as shown in Figure 3. These plots provide a visual representation of the combined effects of the transesterification parameters on biodiesel production and facilitate the identification of optimum operating conditions. The curved surfaces observed in the plots indicate the presence of interaction effects among the investigated variables, which is consistent with the ANOVA results presented in Table 3.

**Figure 3:**
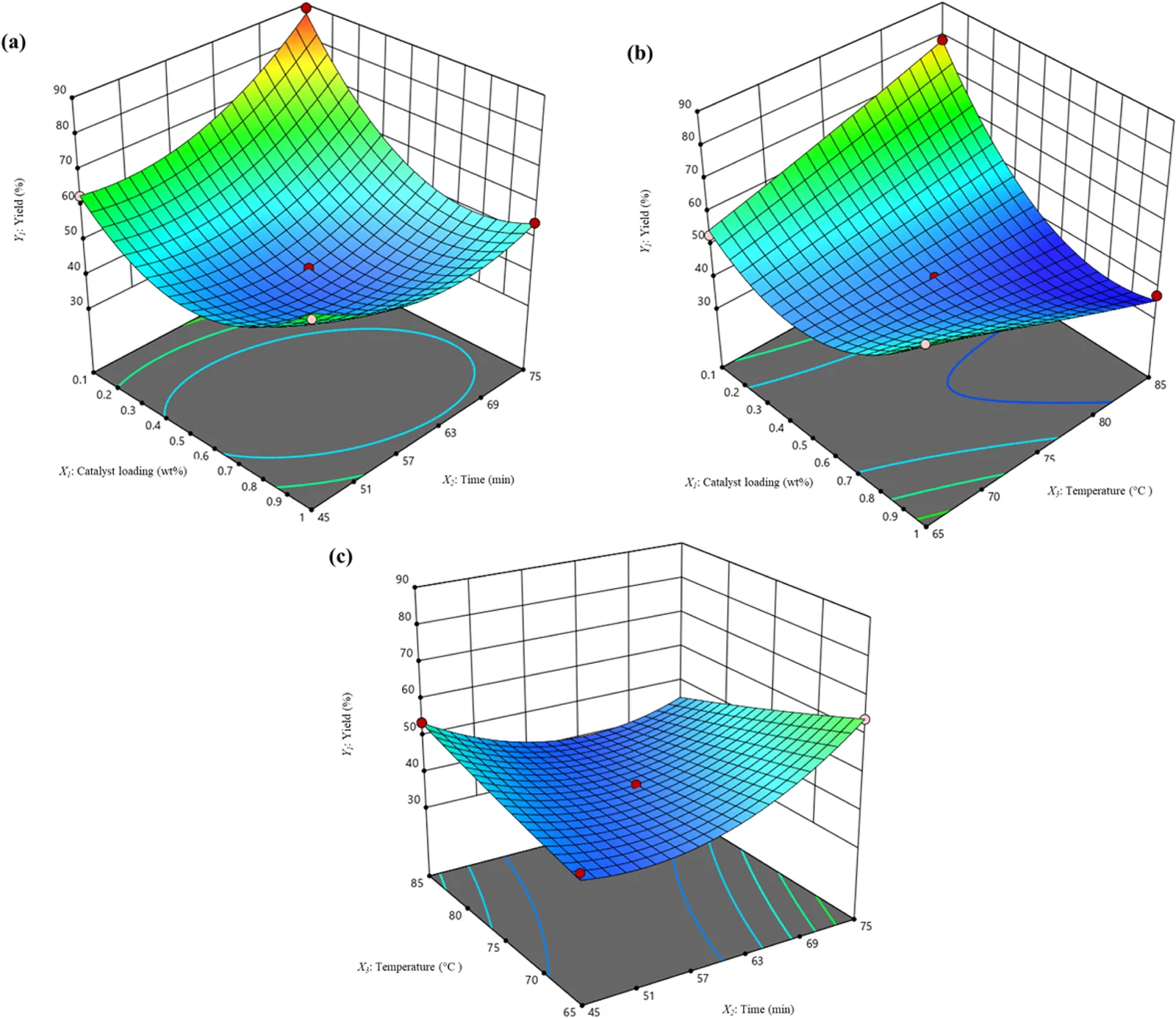
Effects of independent variables on the response of biodiesel yield. (a) Interaction of catalyst loading and reaction time. (b) Interaction of catalyst loading and reaction temperature. (c) Interaction of reaction time and reaction temperature.

Figure 3(a) illustrates the interaction between catalyst loading and reaction time on biodiesel yield. The response surface shows that biodiesel yield increased with increasing reaction time but decreased as catalyst loading increased. The highest biodiesel yield was obtained at low catalyst loading and extended reaction time, indicating that sufficient reaction duration promoted the conversion of lipids into fatty acid methyl esters (FAMEs). However, increasing the catalyst concentration beyond the optimum level resulted in a reduction in biodiesel yield. A similar observation was reported by Farobie et al. (2024), who found that increasing catalyst loading beyond the optimum concentration did not further enhance biodiesel production due to side reactions and increased reaction mixture viscosity, which limited mass transfer between reactants.^21^ Furthermore, it has been reported that an excessive catalyst loading can promote saponification reactions between free fatty acids and alkaline catalysts, leading to soap formation.^22^ The generated soap consumes catalyst, hinders phase separation between biodiesel and glycerol, and ultimately reduces biodiesel recovery and yield. This effect becomes more pronounced when feedstocks contain higher levels of free fatty acids.

The interaction between catalyst loading and reaction temperature is presented in Figure 3(b). Biodiesel yield was maximized at lower catalyst concentrations and moderate reaction temperatures. As catalyst loading increased, a noticeable reduction in yield was observed, particularly at higher temperatures. Excessive catalyst concentrations may promote side reactions, while elevated temperatures can lead to methanol evaporation and reduced availability of alcohol for transesterification. The pronounced curvature of the response surface further indicates a strong interaction between catalyst loading and temperature, which agrees with the ANOVA results where the *X*_1_ *X*_3_ interaction term exhibited the highest *F*-value among all interaction terms.

Figure 3(c) shows the combined effect of reaction time and reaction temperature on biodiesel yield. The response surface indicates that increasing reaction time generally enhanced biodiesel production, suggesting that prolonged reaction periods allowed greater conversion of triglycerides into methyl esters. In contrast, increasing reaction temperature did not consistently improve biodiesel yield and resulted in lower yields at certain operating conditions. This phenomenon may be associated with methanol loss through evaporation and the occurrence of competing reactions at elevated temperatures. A similar observation was reported by Weldeslase et al., who found that increasing the reaction temperature beyond the optimum level did not enhance biodiesel conversion and could reduce biodiesel production due to methanol evaporation at elevated temperatures.^23^ Therefore, an appropriate balance between reaction time and reaction temperature is required to achieve efficient biodiesel conversion. In this work, the response surface analysis demonstrated that catalyst loading was the most influential factor affecting biodiesel yield, followed by reaction time, whereas reaction temperature exhibited a comparatively smaller individual effect.

### 3.3 Model Optimization and Validation

The optimization of transesterification parameters was performed using the numerical optimization function of Response Surface Methodology (RSM) to maximize biodiesel yield from *Ulva lactuca* lipids. The optimum operating conditions predicted by the developed quadratic model are presented in Table 5. The model suggested that a catalyst loading of 0.10 wt %, reaction time of 75 min, and reaction temperature of 75 °C would produce the highest biodiesel yield, with a predicted value of 87.35%. To validate the adequacy of the developed model, an experiment was conducted under the optimum conditions predicted by RSM. As shown in Table 5, the experimental biodiesel yield obtained was 89.00%, which was in close agreement with the predicted yield of 87.35%. The small deviation between the experimental and predicted values indicates that the developed model was capable of accurately describing the transesterification process and predicting biodiesel yield within the investigated experimental range.

**Table 5:** Validation of the Optimal Value for *Ulva lactuca* Transesterification

Optimal condition	Catalyst loading	Time	Temperature	Experimental value	Predicted value	Std deviation	Percentage error (%)
Yield (%)	0.10 wt %	75 min	75 °C	89.00%	87.35%	0.70	1.65

The agreement between the experimental and predicted values is further illustrated in Figure 4, where the data points are closely distributed along the diagonal line. This distribution demonstrates a strong correlation between the model predictions and the experimental observations, confirming the reliability of the quadratic regression model. The high predictive capability of the model is also consistent with the coefficient of determination (*R* ^2^ = 0.9943), which indicates that the model adequately represents the experimental data. The optimization and validation results confirm that Response Surface Methodology is an effective tool for predicting and optimizing biodiesel production from *Ulva lactuca* lipids. The validated optimum conditions can therefore be applied to maximize biodiesel yield while minimizing the number of experimental trials required.

**Figure 4:**
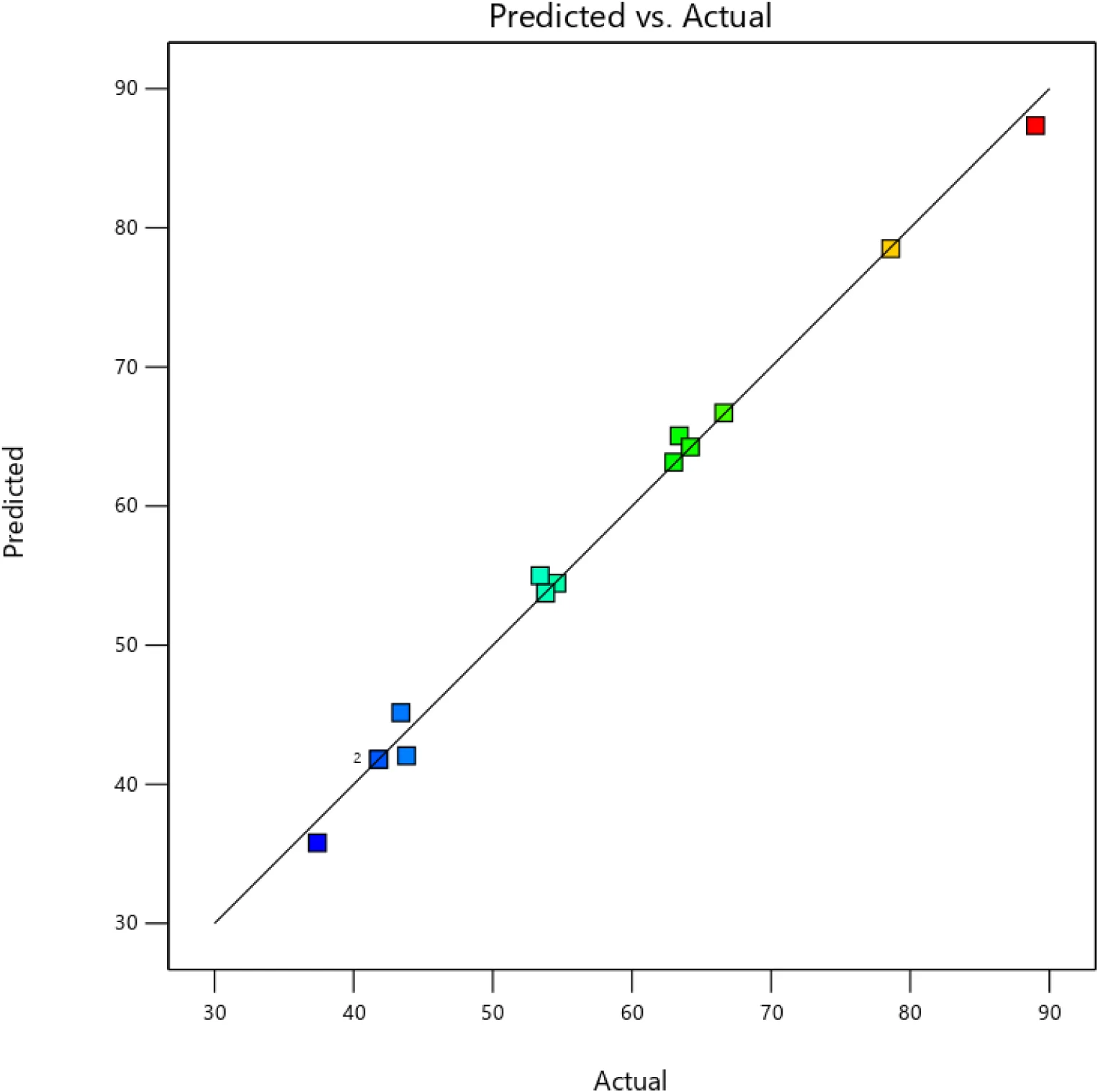
Distribution of predicted values and actual results of transesterification reactions.

### 3.4 Physicochemical Properties of Biodiesel from *Ulva lactuca*

The physicochemical properties of the biodiesel produced from *Ulva lactuca* are presented in Table 6 and evaluated against the ASTM D6751 and EN 14214 specifications. The synthesized biodiesel exhibited a density of 862 ± 0.1 kg/m^3^ at 15 °C, which falls within the acceptable range prescribed by EN 14214 (860–900 kg/m^3^). Fuel density is an important parameter because it influences fuel injection characteristics, atomization behavior and combustion efficiency. The density obtained in this study is comparable to values reported for biodiesel derived from marine macroalgae and other renewable feedstocks, indicating that the transesterification process effectively converted algal lipids into fatty acid methyl esters (FAMEs) with suitable fuel characteristics.^24^ The kinematic viscosity measured at 40 °C was 5.50 ± 0.19 mm^2^/s. This value complied with ASTM D6751 requirements, although it slightly exceeded the upper limit established by EN 14214. The relatively elevated viscosity may be attributed to the substantial proportion of long-chain fatty acid methyl esters such as palmitic acid, present in the FAME profile.^25^ Similar observations have been reported by Farfan-Cabrera et al. (2022),^26^ who demonstrated that biodiesel containing higher concentrations of saturated fatty acid esters generally exhibits increased viscosity. Nevertheless, the measured value remained close to the regulatory threshold and is unlikely to significantly impair fuel handling or combustion performance.

**Table 6:** Physicochemical Properties of *Ulva lactuca* Biodiesel and Comparison with Biodiesel Standards

Properties	Unit	*Ulva lactuca* biodiesel	ASTM D6751	EN 14214
Density (15 °C)	kg/m^3^	862 ± 0.1	-	860–900
Kinematic viscosity (40 °C)	mm^2^/s	5.50 ± 0.19	1.9–6.0	3.5–5.0
Cloud point	°C	–3.5 ± 0.1	–3 to −12	–
Pour point	°C	–8.0 ± 0.2	–15 to −16	–
Flash point	°C	179 ± 2	≥130	≥120
Acid value	mg KOH/g	1.68 ± 0.08	≤0.50	≤0.50
Calorific value	MJ/kg	38.4	–	–
Iodine value	g I_2_/100 g	40.20	–	≤120

The low-temperature performance of the biodiesel was assessed through cloud point and pour point measurements. The cloud point and pour point were determined to be −3.5 ± 0.1 °C and −8.0 ± 0.2 °C, respectively. These values indicate favorable cold-flow behavior and suggest that the fuel can maintain adequate fluidity under relatively low-temperature conditions. The observed cold-flow properties are likely associated with the predominance of monounsaturated fatty acids within the biodiesel composition, which generally contribute to lower crystallization temperatures and improved fuel flow characteristics.^27^ These particular data for the pour point aligned with a similar study by Haghighi et al. (2022),^28^ which recorded a value below 0 °C for biodiesel from *Spirulina* algae oil, mainly associated with the high levels of monounsaturated unsaturated fatty acids (MUFA) present. A low pour point of −8 °C for the biodiesel demonstrated the practicality of usage in regions with low temperatures without any chances of solidifying into wax crystals and losing the fluidity of the fuel. These properties enabled the biodiesel to be more desirable in terms of performance under cold conditions.^29^ The flash point of the produced biodiesel was 179 ± 2 °C, substantially exceeding the minimum requirements specified by ASTM D6751 and EN 14214. A high flash point is desirable because it reflects improved storage and transportation safety as well as the effective removal of residual methanol during purification. The high flash point obtained in this study therefore confirms the satisfactory quality and handling safety of the fuel.

The acid value was measured at 1.68 ± 0.08 mg KOH/g, which exceeded the maximum allowable limit of 0.50 mg KOH/g specified by both ASTM D6751 and EN 14214. Elevated acid values are generally associated with residual free fatty acids (FFAs) and may contribute to fuel degradation, corrosion of engine components, and deposit formation during prolonged use.^30^ Although the initial FFA content of the extracted *Ulva lactuca* lipid was not quantified in this study, residual FFAs may have contributed to the elevated acid value of the resulting biodiesel. This is relevant to KOH-catalyzed transesterification because FFAs can react with the alkaline catalyst and promote soap formation, potentially affecting conversion efficiency and subsequent product purification. Miyuranga et al. (2022)^31^ demonstrated that pretreatment through acid esterification can reduce FFA content and improve biodiesel quality. Therefore, acid-catalyzed esterification prior to alkaline transesterification, together with improved downstream purification, should be considered in future studies to reduce the final acid value. Although the present biodiesel did not meet the acid-value specification, the sustainability potential demonstrated in this study relates primarily to the utilization of *Ulva lactuca* as a renewable, nonfood biomass resource and its optimized conversion into biodiesel. Further improvement in fuel quality, such as reduction of the acid value, is required before practical fuel application. The iodine value was considerably lower than the maximum limit established by EN 14214, indicating a relatively low degree of unsaturation and suggesting favorable oxidative stability during storage. Similar findings have been reported for biodiesel derived for biodiesel derived from third-generation feedstocks, where lower iodine values were associated with enhanced fuel stability and improved combustion characteristics.^32^ The calorific value of the produced biodiesel was 38.4 MJ/kg, which falls within the typical range reported for biodiesel fuels. This value indicates that the *Ulva lactuca* biodiesel possesses an energy content comparable to conventional biodiesel and confirms that the fuel retains a suitable energy density for potential fuel applications.^33^

A quantitative comparison with previous Ulva lactuca-based biodiesel studies provides further context for the present findings. Binhweel et al. (2023)^34^ reported a lipid yield of 7.6 ± 0.10% and an optimized biodiesel yield of 88.77% using a 1:12 oil-to-methanol molar ratio, 1.5 wt % NaOH, a reaction temperature of 63 °C, and a reaction time of 1.7 h. Yameen et al. (2024)^8^ reported a lipid yield of 10.17% and a biodiesel yield of 92.56% using 3.96 wt % Cu-BTC@AC catalyst, a 15:1 methanol-to-lipid ratio, 65 °C, and 140 min with a FAME content of 98.12%. In comparison, the present study obtained a lipid extraction yield of 4.06% and a recovered biodiesel product yield of 89.0% using 0.10 wt % KOH, a methanol-to-lipid mass ratio of 10.8:1, 75 °C, and 75 min. Although the lipid extraction yield was lower than those reported in the previous studies, the recovered biodiesel yield was comparable, while requiring a lower catalyst loading and shorter reaction time. These differences may be attributed to variations in extraction conditions, catalyst systems, alcohol-to-lipid ratios, and transesterification conditions.

## 4 Conclusions

This study demonstrated the production and optimization of biodiesel from *Ulva lactuca* lipids through KOH-catalyzed transesterification using Response Surface Methodology based on a Box-Behnken Design. The developed quadratic model was statistically significant (*p* = 0.0004, *R* ^2^ = 0.9943) with catalyst loading identified as the most influential factor, followed by reaction time, while reaction temperature showed a smaller individual effect within the investigated range. The optimum conditions were 0.10 wt % catalyst loading, 75 min reaction time, and 75 °C reaction temperature, resulting in an experimental biodiesel yield of 89.0% compared with the predicted yield of 87.35%. The produced biodiesel exhibited a density of 862 kg/m^3^, a kinematic viscosity of 5.50 mm^2^/s, a cloud point of −3.5 °C, a pour point of −8.0 °C, a flash point of 179 °C, and an iodine value of 40.20 g I_2_/100 g. While several measured properties complied with the applicable ASTM D6751 and/or EN 14214 specifications, the acid value of 1.68 mg KOH/g exceeded the maximum limit of 0.50 mg KOH/g. In addition, the calorific value of 38.4 MJ/kg was within the typical range reported for commercial biodiesel fuels. Therefore, further improvements in pretreatment and purification processes are required to reduce acidity and achieve full compliance with international biodiesel quality specifications. Overall, the findings demonstrate the potential of *Ulva lactuca* as a nonfood lipid feedstock for biodiesel production while highlighting the need for further improvement in fuel quality before practical application.

## Data Availability

Data sets and analyses from this study can be provided by the corresponding author upon reasonable request. All data in this study are presented in the manuscript.
